# Supplementary figures and images for: The mGluR5 agonist CHPG enhances human oligodendrocyte differentiation
Source: Acta Neuropathol Commun. 2025 Oct 3;13:210. doi: 10.1186/s40478-025-02124-7 (PMC12492762; doi:10.1186/s40478-025-02124-7)

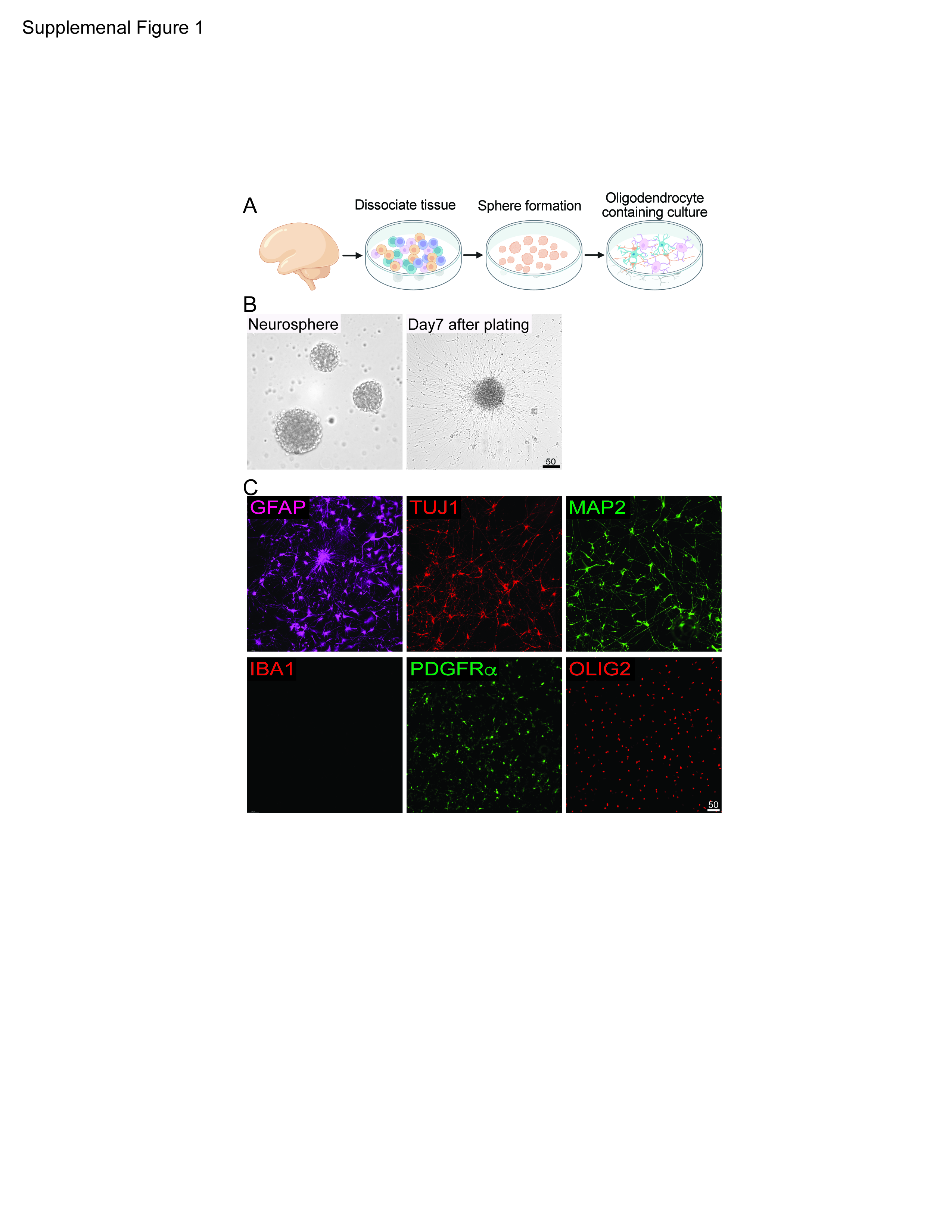

Supplement: Supplementary file 1 — Supplemental Figure 1. Human brain cell culture. (A) Fetal brain tissues (gestational weeks 17 to 19) were dissociated and allowed the formation of neurospheres in PDGF medium (see Methods) for 7-10 days, then plated to allow migration of cells for 4-5 days. (B) On the day of compound treatments, the culture contains GFAP+ astrocytes, TUJ1+, MAP2+ neurons, PDGFRα+, OLIG2+ OPCs. The culture is devoid of IBA1+ microglia. Scale bar = 50 μm. (TIF 36767 KB) [file 40478_2025_2124_MOESM1_ESM.tif]

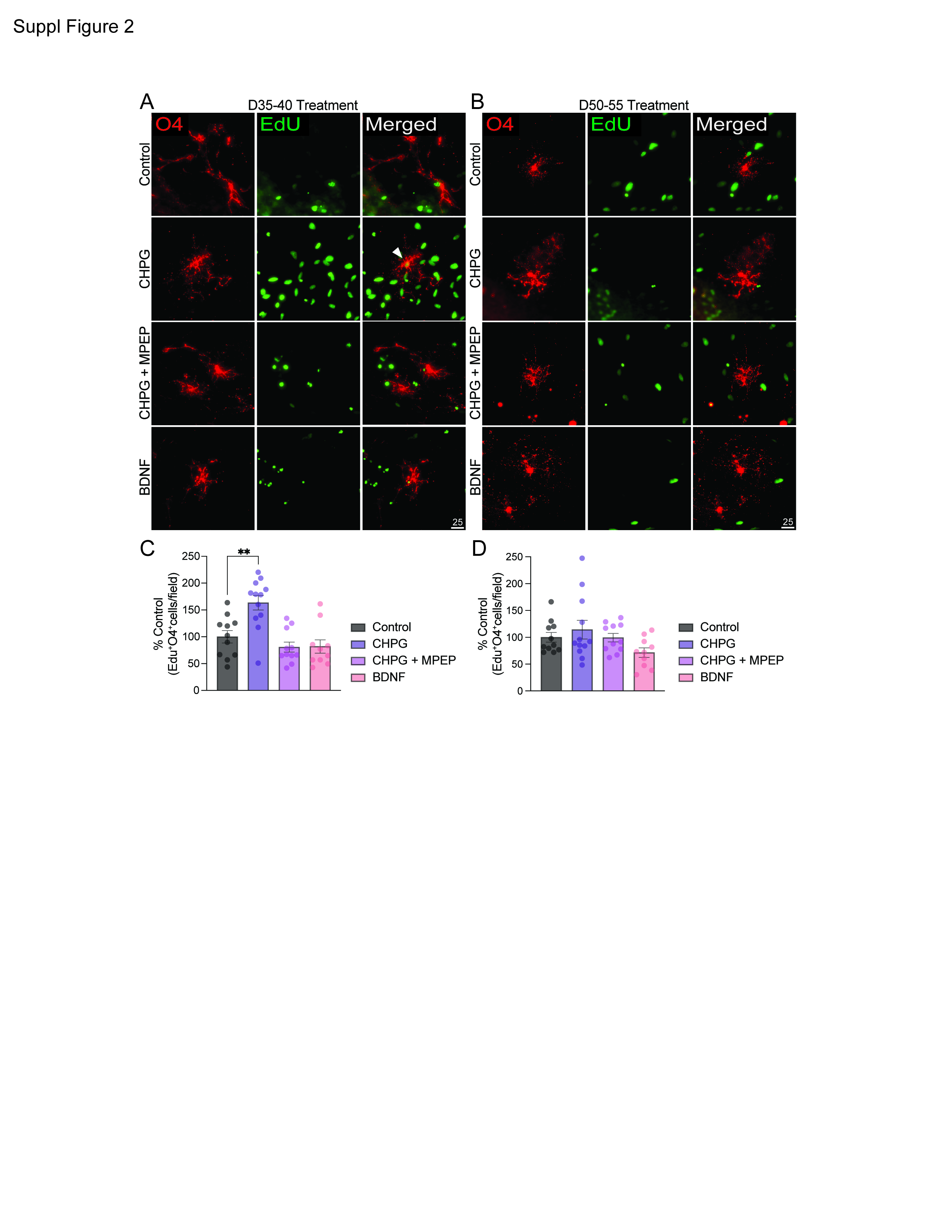

Supplement: Supplementary file 2 — Supplemental Figure 2. CHPG increases proliferation of pre-OLs. Edu was added during pre-OL stage (day 35-40, panel A) or immature-to-mature OL stage (day 50-55, panel B) of iPSC differentiation, concurrently with CHPG (30 μM final concentration), CHPG+MPEP (20 μM final concentration), or BDNF (10ng/ml final concentration) treatment. (C-D) On day 55, oligodendrocytes that proliferated were quantified by Edu detection and co-immunofluorescent staining with O4. CHPG significantly increased proliferation of OL lineage cells only when CHPG was given during pre-OL stage (day 35-40) (N = 3 independent experiments). The effect was reversed by MPEP. Scale bar = 25 μm. Data are expressed as mean ± SEM. (TIF 36113 KB) [file 40478_2025_2124_MOESM2_ESM.tif]

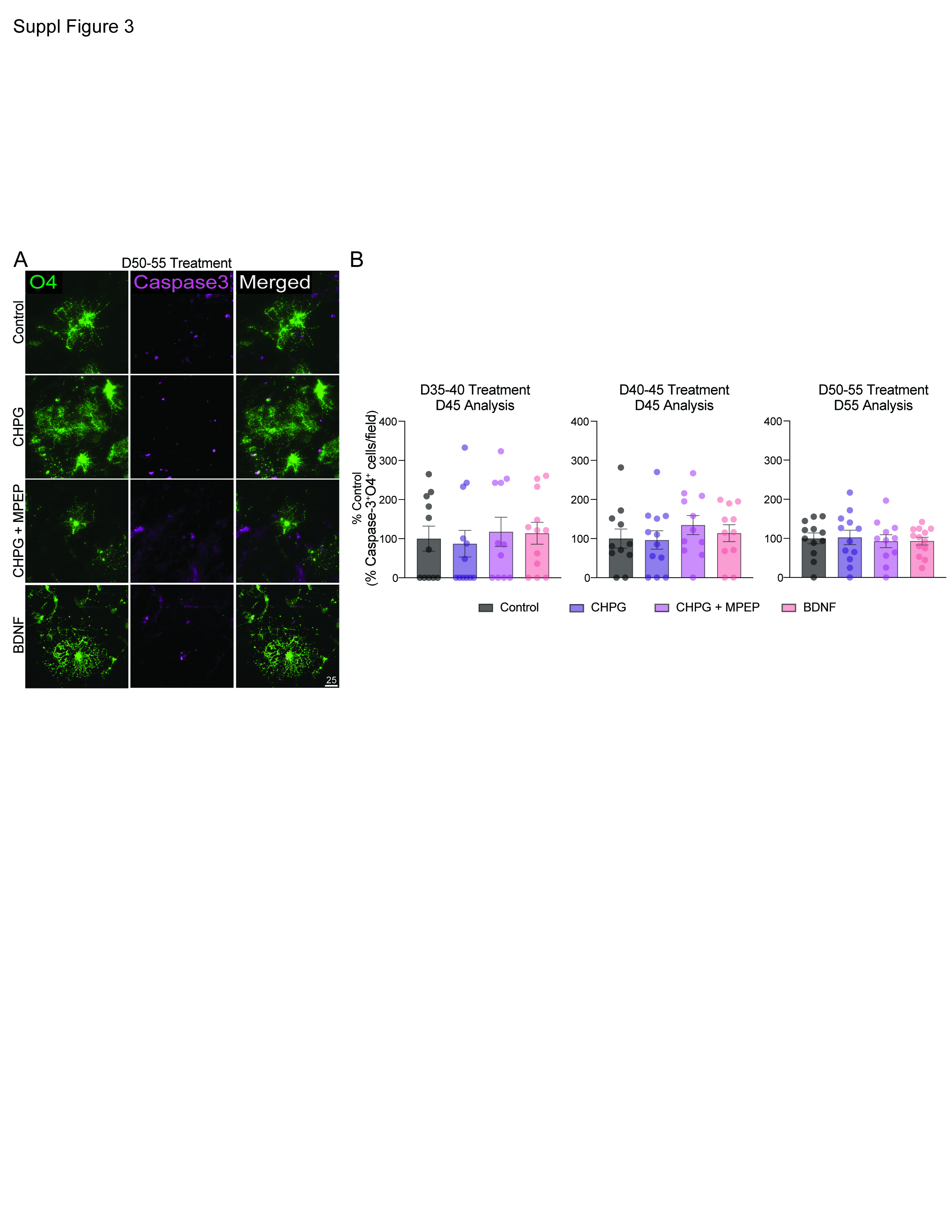

Supplement: Supplementary file 3 — Supplemental Figure 3. CHPG does not affect OL survival. (A) OL cell death was analyzed with Caspase-3 antibody at the end of CHPG (30 μM final concentration) treatment at various stages of iPSC differentiation (day 35-40, 40-45, or 50-55) and colocalized with O4. (B) Overall cell death remained low throughout time points (<4% or all O4+ cells), and no significant difference was observed in response to CHPG, MPEP, or BDNF treatments (N = 3 independent experiments). Scale bar = 25 μm. Data are expressed as mean ± SEM. (TIF 36818 KB) [file 40478_2025_2124_MOESM3_ESM.tif]
