# Supplementary material for: The mGluR5 agonist CHPG enhances human oligodendrocyte differentiation
Source: Acta Neuropathol Commun. 2025 Oct 3;13:210. doi: 10.1186/s40478-025-02124-7 (PMC12492762; doi:10.1186/s40478-025-02124-7)
Supplement: Supplementary file 4 — Supplemental Table 1. List of MS and control tissues used in this study. (DOCX 16 KB) [file 40478_2025_2124_MOESM4_ESM.docx]

| Sample ID | Age | MS status | Sex | RNA Integrity Number | Disease duration (years) | Lesion type |
| --- | --- | --- | --- | --- | --- | --- |
| CO22 | 69 | Control | female | 5.2 | NA | NA |
| CO64 | 63 | Control | female | 6 | NA | NA |
| CO90 | 83 | Control | male | 4.7 | NA | NA |
| PDCO40 | 61 | Control | female | 5.7 | NA | NA |
| MS426 | 48 | MS | female | 7 | 28 | chronic active |
| MS438 | 53 | MS | female | 5 | 18 | chronic active |
| MS513 | 51 | MS | male | 4.8 | 18 | chronic active |
| MS528 | 45 | MS | female | 5.2 | 25 | chronic active |

Supplementary Table 1
